# Serologic Evidence of Orthopoxvirus Infection in Buffaloes, Brazil

**DOI:** 10.3201/eid1804.111800

**Published:** 2012-04

**Authors:** Felipe Lopes de Assis, Graziele Pereira, Cairo Oliveira, Gisele Olinto Libânio Rodrigues, Marcela Menezas Gomes Cotta, Andre Tavares Silva-Fernandes, Paulo Cesar Peregrino Ferreira, Cláudio Antônio Bonjardim, Giliane de Souza Trindade, Erna Geessien Kroon, Jônatas Santos Abrahão

**Affiliations:** Universidade Federal de Minas Gerais, Belo Horizonte, Minas Gerais, Brazil

**Keywords:** Vaccinia virus, Orthopoxvirus, buffaloes, poxvirus, buffalopox virus, viruses, Brazil

**To the Editor:** Since 1999, several exanthematous vaccinia virus (VACV) outbreaks affecting dairy cattle and rural workers have been reported in Brazil (*1**,**2*). VACV, the prototype of the genus *Orthopoxvirus* (OPV), exhibits serologic cross-reactivity with other OPV species and was used during the World Health Organization smallpox eradication campaign (*3*). The origin of VACV in Brazil is unknown, although some studies have suggested that VACV strains used during the campaign may be related to outbreaks of bovine vaccinia (BV) (*2*). In Brazil, BV affects the milk industry and public health services (*1**,**2**,**4**,**5*). During outbreaks, dairy cattle developed lesions on the teats and udders, causing a decrease in milk production (*1**,**2**,**4**,**5*).

Another VACV subspecies, buffalopox virus (BPXV), has been isolated from buffaloes (*Bubalus bubalis*) in rural areas in India and causes clinical signs that resemble those seen during BV outbreaks in Brazil (*6*). Recent genetic analysis of BPXV samples confirmed its close relationship to VACV-like viruses, although each virus has distinct genetic signatures (*1**,**2**,**6*). Until recently, buffalo herds have been almost exclusive to northern Brazil. However, the buffalo market has experienced great expansion in this country, and today, there are herds in all geographic regions of Brazil. These buffalo herds are hypothetically at risk for VACV infection, on the basis of the outbreaks caused by BPXV that have been described in India (*6*). To assess the risk for OPV infection in milk buffaloes in Brazil, we conducted a serosurvey of herds from southeastern Brazil, the region most affected by BV.

During October 2010, we screened milk buffalo herds in rural areas of Minas Gerais State, Brazil. Serum samples were collected from 48 female buffaloes used for milk production; these animals belonged to 3 neighboring properties in Carmo da Mata city (20°33′28′′S, 44°52′15′′W), which is in the same mesoregion where the VACV Passatempo virus strain was isolated during an outbreak in 2003 (*5*). Since then, several outbreaks have been reported in this area.

Serum samples were inactivated, and an OPV plaque-reduction neutralization test (PRNT) was performed (*7*). The serum titer was defined as the highest dilution that inhibited >70% of viral plaques relative to the level of inhibition of the negative controls. Samples also underwent ELISA for OPV IgG as described (*4*). Bovine serum samples were used as positive and negative controls (*1**,**4*). OPV-PRNT specificity (98.4%) and sensitivity (93.5%) were confirmed by using receiver-operating characteristic analysis as described (*8*). The tests were performed in duplicate.

Of the 48 buffalo serum samples, 15 (31.25%) contained neutralizing antibodies against OPV; of these, 6 (40%) had titers of 20, 5 (33.3%) had titers of 40, and 4 (26.6%) had titers >80 (Table). The ELISA yielded results similar to those of the PRNT; of the 48 serum samples, 17 (35.41) were IgG positive (Table). A total of 14 samples were coincident in the PRNT and the ELISA, including most of those with high titers by PRNT. To detect viral DNA, we conducted nested PCR to amplify the viral growth factor gene (*9*) and real-time PCR to amplify the A56R gene (*10*); results were negative for all 48 serum samples.

**Table:** Results of testing for orthopoxvirus seropositivity in milk buffalo herds, Minas Gerais State, Brazil, October 2010*

Test	No. (%) samples
PRNT	
Total positive	15 (31.2)
Titer	
20	6 (40.0)
40	5 (33.3)
80	2 (13.3)
160	2 (13.3)
Total negative	33 (68.7)
ELISA	
Total positive	17 (35.4)
Total negative	31 (64.6)
PRNT and ELISA positive	14 (29.2)

*Serum samples were collected from 48 female buffaloes used for milk production. A positive titer was defined as the highest dilution that inhibited >70% of viral plaques relative to the level of inhibition of the negative controls. Samples also underwent ELISA for orthopoxvirus IgG as described (*4*). PRNT, plaque-reduction neutralization test.

We detected antibodies against OPV in buffaloes in Brazil 10 years after the first reported VACV outbreak in cattle in Minas Gerais State (*1*). Because PRNT and ELISA indicate the presence of OPV antibodies in a nonspecific manner (OPV serologic cross-reaction), it was not possible to determine the species responsible for these results. However, seropositive buffaloes may have been exposed to VACV, the only OPV known to be circulating in Brazil (*1**,**2**,**4**,**5**,**8*).

The management of milk buffaloes in Brazil is similar to that of dairy cows, including manual milking (*1**,**4**,**5*). Cow milkers usually work on >2 farms, and the farm infrastructure commonly is unsophisticated (*1**,**4**,**5*). These conditions were shown to be favorable for the spread of VACV among cattle, which suggests that the same conditions could lead to the introduction of VACV into buffalo herds. Because some BV outbreaks are not reported by the farmers, it is not possible to know exactly how or when a buffalo herd in the study area was exposed to the virus. However, milkers who work with both cattle and buffalo may be a route of viral transmission, although other sources of exposure are possible (*8*). Although no exanthematous VACV outbreaks have been described in milk buffaloes in Brazil, our results suggest that buffalo herds may be exposed to VACV in BV-affected areas and therefore may be at risk for VACV infection. Further research is needed to determine routes of infection, including whether humans working as milkers contribute to virus transmission.
